# Hypertensive Emergency and Atypical Hemolytic Uremic Syndrome Associated with Cocaine Use: A Diagnostic and Therapeutic Challenge

**DOI:** 10.3390/diseases13050153

**Published:** 2025-05-15

**Authors:** Elena Jiménez Mayor, José C. De La Flor, André Rocha Rodrigues, Celia Rodríguez Tudero, Rocío Zamora González-Mariño, Jacqueline Apaza, Esperanza Moral Berrio, Javier Deira Lorenzo

**Affiliations:** 1 Department of Nephrology, Hospital San Pedro de Alcántara, 10001 Cáceres, Spain; andre.rocha@salud-juntaex.es (A.R.R.); javierlorenzo.deira@salud-juntaex.es (J.D.L.); 2 Department of Nephrology, Hospital Central de la Defensa Gómez Ulla, 28047 Madrid, Spain; jflomer@mde.es; 3 Department of Medicine and Medical Specialties, Faculty of Medicine, Alcala University, 28805 Madrid, Spain; 4 Spain Health Sciences Doctoral Program, Faculty of Medicine, Alcala University, 28805 Madrid, Spain; 5 Department of Nephrology, Hospital Universitario de Salamanca, 37007 Salamanca, Spain; crodrigueztudero@usal.es; 6 Surgery Department, Faculty of Medicine, University of Salamanca, 37007 Salamanca, Spain; 7 Department of Nephrology, Hospital Universitario General Villalba, 28400 Madrid, Spain; rocio.zamora@quironsalud.es; 8 Department of Nephrology, Hospital Rey Juan Carlos, 28933 Madrid, Spain; jacqueline.apaza@hospitalreyjuancarlos.es; 9 Department of Nephrology, Hospital Universitari Doctor Josep Trueta, 17007 Girona, Spain; emoral.girona.ics@gencat.cat

**Keywords:** atypical hemolytic uremic syndrome, thrombotic microangiopathy, cocaine, acute kidney injury

## Abstract

Background: Atypical hemolytic uremic syndrome (HUS) is a rare form of thrombotic microangiopathy (TMA) characterized by complement dysregulation. Cocaine use has been reported to be a potential trigger of TMA; however, the underlying mechanisms remain poorly elucidated. Proposed hypotheses include direct endothelial injury, activation of the complement cascade, and the unmasking of whether HUS is genetic or acquired. Case Report: We report the case of a 47-year-old man who presented with hypertensive emergency and acute kidney injury following intranasal cocaine use. The laboratory findings were consistent with microangiopathic hemolytic anemia (MAHA), thrombocytopenia, and markedly elevated lactate dehydrogenase (LDH) levels. Renal biopsy (RB) revealed classic features of TMA, including glomerular capillary thrombosis, fibrinoid necrosis, and acute tubular injury. Complement studies demonstrated reduced levels of Factor I, indicative of complement dysregulation. The patient was treated with therapeutic plasma exchange and four weekly doses of eculizumab, resulting in hematologic remission and significant improvement in renal function, without the need for dialysis. Genetic testing for known atypical HUS-associated mutations was negative; therefore, maintenance therapy with eculizumab was discontinued without clinical relapses. Discussion: This case underscores cocaine as a rare but important precipitating factor for atypical HUS in predisposed individuals. Early diagnosis, RB, and complement evaluation were essential in determining the etiology and guiding targeted therapy. Complement inhibition with eculizumab was effective in halting disease progression and preventing long-term renal damage. Conclusions: This case highlights the relevance of considering cocaine use as a potential trigger of complement-mediated TMA. Early identification of aHUS features and prompt initiation of complement inhibition therapy may be critical to preventing irreversible kidney injury.

## 1. Introduction

Hypertensive emergency is a severe clinical condition that, if not properly treated, can lead to irreversible damage to vital organs such as the brain, kidneys, heart, and retinas. The clinical situations leading to hypertensive emergency are diverse and may be of primary origin, such as uncontrolled hypertension, or secondary to other disorders such as substance use, kidney diseases, or systemic conditions. When hypertensive emergency is accompanied by acute kidney injury (AKI), microangiopathic hemolytic anemia (MAHA), and thrombocytopenia, the differential diagnosis can be challenging. Conditions such as thrombotic thrombocytopenic purpura (TTP), hemolytic uremic syndrome (HUS), and thrombotic microangiopathies (TMAs) secondary to severe vascular damage should be considered [1].

In such cases, a structured clinical approach is essential to guide diagnosis and management. Atypical HUS (aHUS) should be suspected when the following constellation of findings is present: (1) MAHA: elevated lactate dehydrogenase (LDH), reduced haptoglobin, and schistocytes on a peripheral blood smear; (2) thrombocytopenia: typically, <150,000/μL; (3) AKI: elevated creatinine, proteinuria, and/or hematuria; (4) normal or mildly reduced disintegrin and metalloproteinase with a thrombospondin type 1 motif, and member 13 (ADAMTS-13) activity (>10%); (5) the absence of Shiga-toxin-producing *E. coli* infection; (6) evidence of complement dysregulation: low C3 levels, reduced Factor I or H, and/or C3 deposits on a renal biopsy; and (7) the exclusion of other secondary causes of TMA, including autoimmune, infectious, malignant, or drug-induced etiologies.

Early identification of these features is critical, as prompt initiation of complement inhibition therapy can be lifesaving [1]. HUS is a variant of TMA, affecting predominantly the kidney microvasculature. It is classified into two categories: (1) Typical HUS, generally associated with infections with Shiga-toxin-producing bacteria, such as *E. coli* O157:H7, is more common in children and is often preceded by a diarrheal episode, frequently hemorrhagic. (2) aHUS is frequently associated with dysregulation of the alternative complement pathway, leading to uncontrolled activation of the membrane attack complex and, ultimately, endothelial damage. This damage causes microthrombus formation in the renal microcirculation and other organs, resulting in tissue ischemia and the characteristic clinical manifestations of HUS. Furthermore, atypical HUS may be linked to genetic mutations or triggering factors such as infections, autoimmune diseases, certain medications, and, rarely, substance use such as cocaine [1].

Cocaine is a widely used illicit drug that can have multiple adverse effects on various organ systems. Its use has been associated with cardiovascular, neurological, and renal complications. Although the association between cocaine use and aHUS is infrequent, there are reports suggesting a possible relationship [2]. The exact mechanism by which cocaine could induce aHUS has not been fully elucidated, but several hypotheses have been proposed: direct endothelial damage, as cocaine may cause vasoconstriction and direct endothelial injury, which could trigger a TMA [3]; complement activation, as cocaine may induce inappropriate activation of the complement system, like that observed in primary aHUS [3]; and predisposing factors, as individuals with genetic mutations affecting complement regulation may be at higher risk of developing HUS following cocaine use. The clinical manifestations of aHUS associated with cocaine use are like those of any other TMA. Diagnosing aHUS in the context of cocaine use requires high clinical suspicion and the exclusion of other causes of TMA. A detailed history, including substance use history and potential predisposing factors such as mutations in complement-regulatory genes, is essential. The management of aHUS associated with cocaine is complex and must be individualized. Therapeutic strategies should include the cessation of cocaine use, supportive care, and renal replacement therapy if required in cases of severe AKI [3,4,5], as well as complement inhibition with Eculizumab (a monoclonal antibody that inhibits terminal complement activation) [6,7,8,9,10]. However, its use in cocaine-associated cases should be carefully evaluated due to the lack of specific evidence and the high cost of treatment. On the other hand, Plasma exchange can be considered in situations where complement inhibitors are not available or when an autoimmune etiology is initially suspected, as its efficacy in aHUS is limited [11,12,13,14]. The prognosis of aHUS associated with cocaine use depends on multiple factors, including the speed of diagnosis and treatment, the presence of comorbidities, and the degree of renal involvement at the time of presentation. Early detection and appropriate intervention are crucial to improving clinical outcomes and reducing associated mortality [6,7,12,15].

Atypical HUS is a rare entity that can be associated with cocaine use. Although the exact pathophysiology of this association is not fully understood, mechanisms related to endothelial damage and complement activation are postulated. Timely identification and proper management of these cases are essential to improving prognoses and preventing long-term complications. This clinical case, consistent with previously reported real-world observations [15], illustrates the diagnostic and therapeutic management of a patient admitted with hypertensive emergency associated with aHUS triggered by cocaine use.

## 2. Case Report

We present the case of a 47-year-old Caucasian male with no significant past medical history, including no prior diagnoses of hypertension, diabetes mellitus, or dyslipidemia. He reported chronic tobacco use (one pack per day), occasional alcohol consumption, and recreational intranasal cocaine use. Two days after the intranasal administration of 0.5 g of cocaine, the patient presented to the emergency department with progressively worsening binocular visual impairment and a severe headache. On examination, he was disoriented, markedly hypertensive (245/149 mmHg), tachycardic (135 bpm), tachypneic (24 breaths/min), and afebrile (36.8 °C), and maintained an oxygen saturation of 95% on ambient air. No peripheral edema or focal neurological deficits were observed. Electrocardiography demonstrated sinus tachycardia without repolarization abnormalities. Fundoscopic examination revealed grade IV hypertensive retinopathy, characterized by bilateral optic disk edema and cotton wool spots. Initial laboratory investigations demonstrated stage 3 AKI according to the 2021 *Kidney Disease: Improving Global Outcomes* (KDIGO) criteria [4], with a serum creatinine (sCr) level of 4.08 mg/dL and serum urea of 109 mg/dL. Hematologic evaluation revealed thrombocytopenia (platelet count: 94,000/mm^3^) and MAHA, with a hemoglobin level of 6.7 g/dL, LDH of 1142 U/L, and undetectable haptoglobin (<5 mg/dL). Urinalysis revealed microscopic hematuria and 3+ proteinuria on dipstick testing. The patient was admitted to the intensive care unit, where intravenous antihypertensive therapy with labetalol and nitroglycerin was initiated, achieving a reduction in blood pressure to 170/110 mmHg within 48 h. Oral antihypertensive therapy was subsequently optimized with amlodipine, enalapril, and bisoprolol. Cranial computed tomography (CT) excluded acute intracranial pathology, including hemorrhage or mass effect. However, brain magnetic resonance imaging (MRI) demonstrated ischemic changes predominantly in the bilateral subcortical white matter, consistent with microangiopathic injury secondary to malignant hypertension. Peripheral blood smear revealed eight schistocytes per high-power field. The analysis of ADAMTS-13 activity was preserved at 76%. Complement component C3 decreased, while C4 and rheumatoid factors were within the normal limits. Hepatitis B, hepatitis C, and human immunodeficiency virus (HIV) serologies were negative. Serum and urine protein electrophoresis and immunofixation failed to detect monoclonal gammopathy. Plasma and urinary catecholamines and metanephrines were within the normal ranges. Renin, aldosterone, and cortisol levels were also unremarkable. Additional laboratory parameters are summarized in Table 1.

The PLASMIC score was 4, indicating a low likelihood of thrombotic thrombocytopenic purpura (TTP). In view of clinical suspicion for atypical HUS, a percutaneous renal biopsy (RB) was performed.

**Figure 1:**
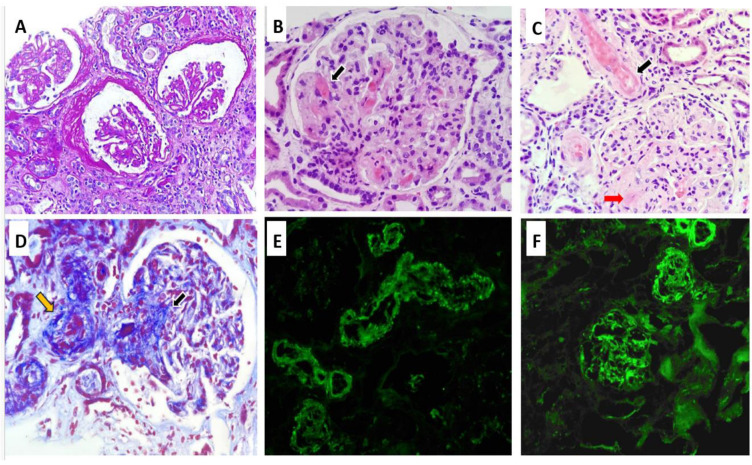
Renal biopsy findings: Ischemic-appearing glomeruli with pericapsular fibrosis, and capillary wrinkling (Periodic acid–Schiff ×20) (**A**). Glomerular capillary wall thickening with luminal obliteration, and fibrinoid necrosis, black arrow (Hematoxylin and Eosin ×40) (**B**). Fibrinoid necrosis involving the arterial wall (black arrow) and glomerular capillary wall (red arrow) (Hematoxylin and Eosin ×40) (**C**). Masson’s trichrome stain highlighting fibrinoid necrosis in glomerular capillaries (black arrow) and arterioles (yellow arrow) (**D**). Focal mesangial C3 deposits by immunofluorescence (**E**). Immunofluorescence showing C3 deposition in vessels walls (**F**).

The RB, as shown in Figure 1, included a total of 12 glomeruli, one of which was globally sclerosed. Several glomeruli exhibited ischemic changes, including tuft retraction, irregular capillary loops, and a widened Bowman’s space. One glomerulus demonstrated segmental sclerosis. In the remaining glomeruli, serial sections revealed a lobulated architecture with markedly thickened capillary walls, almost obliterating the lumina. Features of TMA were evident, including red blood cell fragmentation, fibrinoid necrosis, and focal capillary thrombosis confirmed by positive CD61 immunostaining. Arterioles demonstrated fibrinoid necrosis at the vascular pole, with occasional fragmented erythrocytes within the vessel wall. No associated inflammatory infiltrate was noted. The arteriolar lumina were markedly narrowed, with areas of focal thrombosis and focal subintimal myxoid change. A small artery exhibited concentric intimal thickening with an “onion-skin” configuration. Tubular structures showed evidence of acute tubular injury, including epithelial cell sloughing into the lumen, intraluminal hemorrhagic material, and hyaline casts. Focal tubular atrophy with patchy chronic interstitial inflammation was also observed. Immunohistochemical staining for CD61 was positive in the arterioles and in several glomerular capillaries. Direct immunofluorescence (IF) revealed no glomerular deposits of IgG, IgA, IgM, C1q, kappa, or lambda light chains. Only segmental mesangial staining for C3 (1+) was identified. Vascular staining showed focal fibrinogen positivity along with segmental trapping of IgM, C3, and C1q. Electron microscopy (EM) was not performed due to insufficient sample availability.

Based on the clinical, analytical, and histological findings from light microscopy (LM) and IF, without EM data, the diagnosis was TMA with glomerular capillary obliteration, fibrinoid necrosis in arterioles, and acute tubular damage. Considering these findings and given the severity of the clinical presentation and the decreased levels of Factor I, treatment was initiated with glucocorticoids (prednisone 40 mg/day), and the use of Eculizumab was requested. However, before its administration and while awaiting approval, four early sessions of plasmapheresis were performed due to the severity criteria. The patient received eculizumab four weekly doses of 900 mg, then maintenance doses of 1200 mg every two weeks. One month after admission, the patient exhibited a favorable hematologic response, with progressive stabilization of hemoglobin and platelet counts. Peripheral blood smear analysis demonstrated a reduction in schistocyte percentage from 8% to 5% over 21 days. With effective control of blood pressure and hemolysis, improvements were observed in both retinal findings and renal function, with serum creatinine decreasing to 1.9 mg/dL. Notably, renal replacement therapy (RRT) was not required at any point during hospitalization.

Maintenance therapy with eculizumab was initially continued due to the presumptive diagnosis of complement-mediated TMA. However, upon receiving the complement genetic study results—which revealed no pathogenic variants—treatment was discontinued. The patient has remained clinically stable since the cessation of therapy.

**Figure 2:**
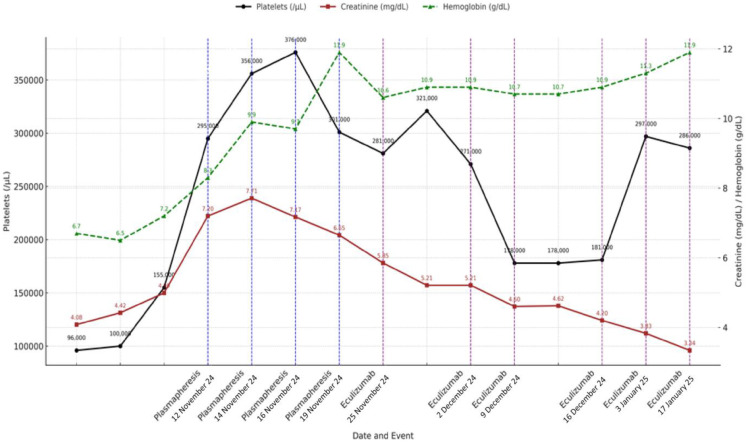
Evolution of platelet count, hemoglobin level, and serum creatinine over time showing key clinical interventions, including therapeutic plasma exchange (blue dashed lines) and eculizumab administration (purple dashed lines). Analytical values are annotated on each data point. Platelet count is represented on left *Y*-axis, while hemoglobin and creatinine levels are plotted against right *Y*-axis. Dates on *X*-axis correspond to timing of therapeutic events.

In summary, the patient received initial treatment with corticosteroids (40 mg/day prednisone with progressive tapering after 2 weeks and discontinuation at 12 weeks), four sessions of plasma exchange, and a complete eculizumab induction regimen (900 mg weekly for four weeks), followed by two rounds of maintenance therapy (1200 mg every two weeks) until it was discontinued after a negative complement genetic workup. The clinical course and laboratory response during hospitalization and follow-up are summarized in Figure 2.

## 3. Discussion

This clinical case represents an uncommon presentation of aHUS, triggered by hypertensive emergency in the setting of recent intranasal cocaine use. Two hypotheses may explain the clinical picture: either cocaine induced malignant hypertension, which then triggered TMA, or cocaine directly triggered complement dysregulation, leading to aHUS, with hypertension as part of its presentation. Despite the absence of identified genetic mutations in our patient, aHUS remains a plausible diagnosis, as complement-mediated TMA can manifest without detectable genetic alterations [6,15]. Previous cases have documented cocaine-associated TMA in both genetically susceptible individuals [3] and those without identifiable mutations, likely through mechanisms involving endothelial injury, vasoconstriction, and possible complement activation [4]. Given the severity of organ involvement and the limited response to blood pressure control alone, the second hypothesis (cocaine-triggered aHUS) appears more likely in our case.

The differential diagnosis initially considered thrombotic thrombocytopenic purpura (TTP) and other TMAs, but these were excluded based on preserved ADAMTS-13 activity (76%) and the absence of secondary triggers such as infection, autoimmune disease, or monoclonal gammopathy. Anticardiolipin IgG/IgM and anti-β2 glycoprotein I antibodies were within the normal limits, further excluding antiphospholipid syndrome (APS) as a potential cause. A negative immunological workup, unremarkable viral serologies, normal serum protein electrophoresis, and a low PLASMIC score further supported the diagnosis of aHUS. Complement studies revealed decreased serum levels of Factor I, consistent with complement dysregulation [2,3]. Elevated classical pathway activity (169%) likely reflects systemic inflammation and immune activation secondary to hypertensive crisis and acute endothelial damage. Although not specific to aHUS, classical pathway activation can occur in acute-phase responses, where heightened immune surveillance and complement engagement are part of the host defense. Additionally, the patient exhibited elevated inflammatory markers such as CRP, which further supports the presence of a pro-inflammatory state potentially contributing to this finding [1].

Unlike typical TMA presentations, this case featured a distinctive trigger—cocaine use. The clinical picture was characterized by neurological impairment, malignant hypertension, AKI, and laboratory findings compatible with MAHA and thrombocytopenia. In comparison with the case reported by Bongetti et al. [4], involving a genetically predisposed patient who developed aHUS after cocaine smoking, our patient demonstrated more severe hemodynamic instability and neurological dysfunction. Likewise, Filho et al. described cocaine-induced renal TMA in a patient presenting with acute tubular necrosis, underscoring how different routes of cocaine administration may elicit distinct clinical phenotypes [4]. These comparisons highlight the clinical variability of cocaine-associated TMA and contextualize the acute severity seen in our patient.

On admission, the patient exhibited profound clinical deterioration, severe headache, acute vision loss, and marked neurological disorientation. These symptoms, together with a hypertensive emergency of 245/149 mmHg and evidence of end-organ damage, reflected critical hemodynamic collapse. The hypertensive crisis likely induced widespread endothelial stress and vascular injury, contributing both to systemic manifestations and to the activation of the complement cascade, thereby promoting thrombotic microangiopathy. This hemodynamic insult, compounded by complement activation, reinforces the dual pathogenic role of malignant hypertension in aHUS [1,2,5,6].

The laboratory evaluation revealed severe renal dysfunction (sCr 4.08 mg/dL), markedly elevated LDH (1142 U/L), undetectable haptoglobin, moderate normocytic anemia, and significant thrombocytopenia. These findings are consistent with previously reported cases of aHUS, although the severity of renal dysfunction and degree of hemolysis in our patient may indicate a particularly aggressive course or a potential delay in diagnosis [6,7].

RB revealed classic TMA features: glomerular capillary obliteration, fibrinoid necrosis, and arteriolar thrombosis. Direct immunofluorescence demonstrated no immune complex deposition, except for segmental mesangial C3 staining. These findings are consistent with drug-induced aHUS, where prominent endothelial injury occurs in the absence of immune deposit, supporting a complement-mediated rather than immune complex-driven mechanism [4].

Eculizumab has revolutionized the treatment of aHUS and is now considered the first-line therapy in most clinical guidelines and expert recommendations [8,9]. In our patient, its use was justified by the severity of the clinical presentation, histological evidence of TMA, and markedly reduced levels of Factor I, strongly suggesting complement dysregulation—even before the results of genetic testing were available. While the patient initially received four plasma exchange sessions due to diagnostic uncertainty and logistical delays, eculizumab was promptly administered thereafter. This approach aligns with the protocol proposed by Cordero et al., consisting of four weekly doses as an induction regime [15]. Remarkably, the drug was discontinued without clinical relapses, in concordance with the favorable outcomes reported in the cohort by Cordero et al. [15].

Furthermore, based on current evidence, Fakhouri and colleagues [8] have proposed a pathophysiology-driven model to guide eculizumab use in secondary aHUS. According to this framework, complement blockade is justified when there is direct evidence of complement dysregulation, as in our case. Even when the trigger is an external factor—such as drugs or infections—treatment is considered appropriate if TMA persists despite resolution of the initiating factor. In this case, persistent hemolysis, renal dysfunction, and biopsy-confirmed TMA—despite the control of blood pressure and cessation of drug exposures—support this rationale. Importantly, our patient’s presentation is highly comparable to the secondary aHUS cases included in the Cordero et al. study, both in terms of severity and laboratory features. This reinforces the justification for initiating eculizumab in this context.

In this case, plasma exchange was initially employed due to the severity of clinical presentation and while awaiting access to eculizumab. Although its benefit in genetically driven aHUS is limited, plasma exchange remains a reasonable option in cases of diagnostic uncertainty or when TTP cannot be definitively excluded. Additionally, it may transiently reduce complement activation during the acute phase, particularly in patients with anti-CFH autoantibodies [10,11]. When used in conjunction with eculizumab, plasma exchange may help achieve earlier disease stabilization in emergent settings [13,14].

The prognosis of aHUS is influenced by factors such as the timing of treatment initiation, the degree of initial renal impairment, and the presence of complement gene mutations. Cohort studies report an improvement in five-year ESRD-free survival from 39.5% to 85.5% with eculizumab [8]. Our patient recovered renal function without requiring dialysis and remained stable at the six-month follow-up after eculizumab discontinuation.

Emerging evidence suggests that patients presenting with malignant hypertension and TMA may harbor mutations in genes regulating the alternative complement pathway, thereby placing them within the spectrum of complement-mediated TMA or primary aHUS. However, it remains unclear whether complement activation in these cases reflects a transient response to external triggers or a manifestation of an underlying genetic predisposition. This ambiguity highlights the need for comprehensive complement gene testing, especially in patients without identifiable secondary causes [16]. In contrast to previously reported cases, the clinical episode described herein was temporally associated with recent cocaine use, strongly suggesting a clear environmental trigger of complement activation in a genetically susceptible host.

To our knowledge, this clinical scenario—characterized by the convergence of cocaine exposure, hypertensive emergency, and complement-mediated TMA—is rarely documented in the literature. Although direct causality is difficult to establish, the temporal association and the exclusion of alternative etiologies strongly support a pathogenic role for cocaine in this context. Our case contributes to the evolving understanding of aHUS phenotypes and underscores the importance of recognizing rare but clinically relevant triggers.

Although the patient exhibited sustained clinical and laboratory improvement, a post-treatment renal biopsy was not performed. While histologic confirmation of recovery would have provided additional insight, it was deemed unnecessary given the favorable trajectory.

## 4. Conclusions

This case underscores the complexity of managing hypertensive emergencies associated with TMA, which may be precipitated by triggers such as cocaine use or malignant hypertension. These factors can induce transient dysregulation of the alternative complement pathway, thereby complicating the diagnosis of aHUS. In this context, comprehensive complement genetic testing is of paramount importance for accurate diagnosis and prognostic stratification. Moreover, this case illustrates the importance of a multidisciplinary approach, with the collaboration of Nephrology, Hematology, Neurology, and Ophthalmology, as well as the use of advanced diagnostic tools, such as renal biopsy and genetic analysis, to guide treatment and avoid subsequent complications.

## Figures and Tables

**Table 1:** Analytical parameters upon admission.

		Normal Values–Unit
Hb	6.7	12–16 g/dL
Platelet count (Plt)	94,000	150–450 × 10^3^/µL
Leukocytes	11.58	3.8–11 × 10^3^/µL
Mean platelet volume (MPV)	12.5	7–10.5 fL
Reticulocyte count	5.2	2–4%
LDH	1142	120–246 U/L
Coombs test	Negative	NA
Total bilirubin	0.8	0.2–1.3 mg/dL
Indirect bilirubin	0.3	0–1.1 mg/dL
Haptoglobin	5	30–200 mg/dL
Antistreptolysin O antibodies	Negative	<20
Total protein (TP)	5.4	6.4–8.7 g/dL
Serum albumin (Alb)	3.2	3–5.5 g/dL
GOT	38	5–32 IU/L
GPT	12	5–33 IU/L
CK	99	46–171 IU/L
CRP	48.7	<0.5 mg/dL
NT-proBNP	789	<300 pg/mL
Serum urea (sU)	100	17–60 mg/dL
Serum creatinine (sCr)	4.08	0.7–1.2 mg/dL
Serum sodium (Na)	136	135–145 mmol/L
Serum potasium (K)	3.5	3.5–5.5 mmol/L
Serum clorum (Cl)	105	95–110 mmol/L
Serum magnesium	1.94	1.7–2.2 mg/dL
HbsAg	Negative	NA
HCV-Ab	Negative	NA
HIV	Negative	NA
Serum cortisol	11.1	4.8–19.5 μg/Dl
Renin concentration	1.5	mUI/L
Serum aldosterone levels	10	pg/mL
Aldosterone-to-renin ratio (ARR)	6.7	0–2.5
Urinary metanephrines	0.4	0–1 mg/24 h
Hydroxyindoleacetic Acid (5-HIAA)	3.3	2–6 mg/24 h
Urinary vanillylmandelic acid (VMA)	4,1	0–13.6 mg/24 h
Complement factor I (CFI)	4	15–50 U/mL
Complement pathway regulation: - Classical pathway - Lectin pathway - Alternative pathway	169% 8.7% 78.72%	69–129% 0–125% 30–113%
CH50	44	40–90 U/ML
CFH levels	20	12–56 mg/dL
Autoantibodies CFH	Undetectable	<18 AU/L
C3 nephritic factor (C3NF)	1.2	Ratio > 1.022
C3	79	90–180 mg/dL
C4	25.9	10–40 mg/dL
Complement C1q	20	15–25 mg/dL
RF	<10	<15 IU/mL
ANA, anti-ds-DNA, ANCA, and cryoglobulin	Negative	NA
Anti-GBM	Negative	<1 AI
Anti-PLA2R Ab (ELISA)	Negative	NA
Anticardiolipin IgG Anticardiolipin IgM	6 5	<12 GPL <12 MPL
Anti-beta-2 glycoprotein IgG Anti-beta-2 glycoprotein IgM	8 10	<20 SGU <20 SMU
IgG	1100	800–1600 mg/dL
IgA	210	70–400 mg/dL
IgM	110	90–180 mg/dL
β2-microglobulin	4	0–20 mg/dL
24 h urine total protein excretion	1.4155	<0.15 g/24 h
Urine red blood cells	4–5%	Undetectable
SPEP/SIFE	No monoclonal spike detected	NA
UPEPE/UIFE	No monoclonal light chains	NA
FLC κ	12.4	4.90–13.70 mg/L
FLC λ	10.9	7.60–19.50 mg/L
FLC κ/λ	1.1	neg
INR	1.26	0.79–1.2
Summary of mutations (genetic study panel 14 genes-SHUa): CFHR 1,2,3,4,5: not detected C3: not detected CFI: not detected MCP: not detected CFB: not detected THBD: not detected DGKE: not detected CFP: not detected ADAMTS13: not detected		

NA: not applicable; WBC: white blood cells; GOT: glutamate-oxaloacetate transaminase; GPT: glutamate pyruvate transaminase; Na: sodium serum; K: potassium serum; Cl: chloride serum; CRP: C-reactive protein; ESR: erythrocyte sedimentation rate; C3: Complement 3; C4: Complement 4; RF: rheumatoid factor; ANA: antinuclear antibody; Anti-ds-DNA: anti-double-stranded DNA antibody; ANCA: anti-neutrophil cytoplasmic autoantibody; Ig: immunoglobulin; SPEP: serum protein electrophoresis; SIFE: serum immunofixation electrophoresis; UPEP/UIFE: urine protein electrophoresis/urine immunofixation electrophoresis; FLC: free light chain; κ: kappa; λ: lambda; RBC/HPF: red blood cell/high-power field; CFH: complement factor H; CFHR: complement factor H-related proteins; CFI: complement factor I; CFP: complement factor P; DGKE: diacylglycerol kinase; MCP: Membrane Cofactor Protein.

## Data Availability

No new data were created or analyzed in this study. The data used to support the findings of this study are available from the corresponding author on request (contact elena.jimenezm@salud-juntaex.es (E.J.M.)).
